# Alcohol use and environmental factors: A cross-sectional study exploring health risks and social implications among Myanmar migrant workers

**DOI:** 10.1371/journal.pone.0343825

**Published:** 2026-03-05

**Authors:** Kanit Hnuploy, Kittipong Sornlorm, Sameh Eltaybani, Nirachon Chutipattana

**Affiliations:** 1 Faculty of Science and Technology, Suratthani Rajabhat University, Surat Thani, Thailand; 2 Faculty of Public Health, Khon Kaen University, Khon Kaen, Thailand; 3 Global Nursing Research Center, Graduate School of Medicine, The University of Tokyo, Tokyo, Japan; 4 Department of Community Public Health, School of Public Health, Walailak University, Nakhon Si Thammarat, Thailand; 5 Excellence Center on Public Health Research: EC for PHR, School of Public Health, Walailak University, Nakhon Si Thammarat, Thailand; Tsinghua University, CHINA

## Abstract

Alcohol use poses unique challenges for migrant workers. Existing literature is limited in understanding how various determinants interact to influence health, family relationships, and social outcomes in this population. This study aims to determine the prevalence of alcohol-related impacts and examine relationships between alcohol use, environmental factors, and their broader effects among Myanmar migrant workers in Thailand. This cross-sectional study surveyed 610 Myanmar migrant workers in Thailand (Sep 2023–Mar 2024) using multi-stage sampling approach. Paper-based questionnaires were utilized, and alcohol use was assessed using the Alcohol Use Disorders Identification Test (AUDIT), with scores categorized as low-risk (0–7), risky (8–19), and probable dependence (≥20). Alcohol-related outcomes were also assessed, and generalized linear mixed models identified associated factors. Of the 610 participants, the majority were male (73.93%), with a mean age of 34.80 years (SD = 10.61) and an age range of 18–73 years. The study found that 38.20% reported a moderate level of alcohol-related impact (95% CI: 34.41–42.12), while 0.82% reported a high level (95% CI: 0.34–1.95). While alcohol addiction significantly increased adverse outcomes (AOR = 1.70, 95% CI: 1.61–1.79), environmental and occupational factors demonstrated stronger associations, with rural residency being the strongest correlate (AOR = 6.52, 95% CI: 4.35–9.77), followed by housing problems (AOR = 5.00, 95% CI: 2.70–9.24). Other significant factors included longer work hours (AOR = 2.39, 95% CI: 1.02–5.60), daily work schedules (AOR = 2.39, 95% CI: 1.64–3.49), poor sleep quality (AOR = 2.09, 95% CI: 1.51–2.90), moderate/poor health (AOR = 2.11, 95% CI: 2.02–2.22), and strained co-worker relationships (AOR = 1.89, 95% CI: 1.88–1.90). These findings highlight that environmental and occupational factors are more strongly associated with alcohol-related impacts than alcohol dependence itself, supporting public health strategies that address structural conditions alongside individual-level interventions.

## Introduction

Alcohol consumption represents a significant global public health challenge, associated with over 200 health conditions including chronic diseases, mental health disorders, and infectious diseases [1,2]. The consequences extend beyond individual health to encompass family disruption through domestic violence, economic instability, and developmental harm to children [3]. At the societal level, alcohol use contributes substantially to injuries, road accidents, reduced workplace productivity, and unemployment, representing a critical public health priority requiring coordinated intervention [1,4].

Migrant worker populations demonstrate particular vulnerability to alcohol-related impacts, with consumption patterns influenced by regional and cultural contexts. Global epidemiological data indicate that 89.2% of international workers consume alcohol, with 5.8% engaging in heavy drinking patterns [5]. A study conducted in Chiang Mai Province, Northern Thailand, revealed that 40.8% of Myanmar migrant workers reported alcohol consumption [6]. This prevalence is higher than the 32% reported among Thai adults in the southern region of the country (Surat Thani Province) [7], and is also higher than the 38.2% reported among adults residing in Myanmar [8].

The present study specifically focuses on Myanmar migrant workers in Thailand for several reasons. They constitute the largest migrant group in Thailand, comprising 78.3% of all migrant workers from Cambodia, Laos, and Vietnam [9]. Within our study provinces (Songkhla and Surat Thani), this population exceeds 101,623 individuals [10]. Crucially, this demographic includes both legally registered and undocumented workers, rendering them a highly vulnerable group with unique risks for alcohol-related impacts due to precarious socio-legal status, limited support systems, and challenging working conditions. Despite their significant presence and elevated risks, limited evidence exists on how environmental and social factors specifically shape these outcomes in this group.

Thailand, as the primary destination for Myanmar migrant workers, reports the highest alcohol consumption rates within the WHO South-East Asia Region [11]. The economic burden attributable to alcohol reaches an estimated 4.3 billion USD annually, representing approximately 1% of the country’s Gross Domestic Product, with healthcare costs accounting for 119.3 million USD of this substantial economic impact [12].

The migration flow stems primarily from economic hardship and political instability in Myanmar, pushing workers toward employment in Thailand’s construction, manufacturing, and agricultural sectors [13,14]. Current demographic data show male Myanmar migrant workers substantially outnumber females across both formal and temporary regularization channels [9]. These workers predominantly find employment in “3D” sectors (Dangerous, Difficult, Dirty) and typically reside in temporary arrangements that undermine family stability and social support networks [13,15].

Despite their economic contributions, migrant workers face hazardous conditions marked by exploitation, abuse, and wage violations [16–18]. They experience multiple behavioral health risks beyond alcohol consumption, including elevated smoking prevalence and physical inactivity rates that contribute to increased overweight/obesity and musculoskeletal disorders [6,19–21]. Significant healthcare access barriers stemming from legal, financial, and linguistic obstacles often force workers to resort to self-treatment, exacerbating health conditions [22–24].

Environmental factors significantly influence alcohol-related behaviors, with housing instability and alcohol outlet density associated with increased harms [25]. Social relationships and peer influences substantially shape drinking patterns, while lower socioeconomic status consistently correlates with harmful alcohol use among migrant populations [26,27]. The Social Determinants of Health framework provides a comprehensive approach for understanding how these structural and intermediary factors collectively create health inequities [28].

While previous research has examined various factors in isolation, limited investigation exists regarding how socio-demographic, economic, labor, health, and environmental determinants interact to collectively shape alcohol-related outcomes [6,29,30]. Addressing this critical gap, this study aims to determine the prevalence of alcohol-related impacts and examine the complex relationships between alcohol use, environmental factors, and their broader effects among Myanmar migrant workers in Thailand. The findings will inform targeted interventions for government agencies, non-governmental organizations, and employers working to reduce alcohol-related impacts in this vulnerable population through evidence-based policies and programs

## Methods

### Research design, sampling and participants

This study employed a cross-sectional design. The target population comprised Myanmar migrant workers in Thailand. Inclusion criteria were: (i) Myanmar nationality; (ii) age ≥ 18 years; (iii) employment in southern Thailand for ≥3 months; (iv) verbal communication ability in Thai or Burmese; (v) willingness to participate; and (vi) being male or female. Exclusion criteria included acute severe physical/mental conditions requiring immediate hospitalization or intensive care (e.g., uncontrolled cardiovascular disease, active psychosis, or severe substance withdrawal), as these could compromise informed consent or safe participation.

The sample size was calculated using Hsieh’s formula for multivariable logistic regression [31,32] yielding 610 participants. Three-stage multistage sampling employed stratified random sampling for province selection, proportional allocation for sample sizes, and respondent-driven sampling (RDS).

Stage 1 (Stratified Provincial Sampling): Southern Thailand was stratified into upper and lower regions (7 provinces each). A simple random sample of one province was then selected from each stratum via lottery method: Surat Thani (upper south) and Songkhla (lower south).

Stage 2 (Proportional Allocation): Based on official migrant population data (April 2023), the sample was allocated proportionally: Songkhla (n = 240; 39,990 migrants) and Surat Thani (n = 370; 61,633 migrants).

Stage 3 (Field Recruitment – RDS Implementation): Within each province, recruitment and data collection were carried out by trained bilingual research assistants (Thai-Burmese) who were independent of any organizational influence. These assistants, all male and aged 35–45 years, had completed upper secondary education. Crucially, they possessed at least 5 years of data collection experience with NGOs, including specific experience in health-related data collection among Myanmar migrant workers. They received comprehensive training which included specialized instruction on respondent-driven sampling (RDS) methodology, ethical recruitment practices, and data collection protocols. Furthermore, the research team provided training on data collection procedures, recording, reporting, and human research ethics according to international standards, encompassing principles of Good Clinical Practice (GCP) to ensure the rights, safety, and well-being of research participants were protected, and that interview data were reliable, accurate, and of high quality.

The RDS process began with the identification of initial “seed” participants using a direct, community-based approach. After conducting preliminary reconnaissance to locate areas with a significant presence of Myanmar migrant workers (e.g., industrial factories, rubber plantations, or construction sites), bilingual research assistants approached individuals in these locations and initiated contact in Burmese. If the individual responded in Burmese, this confirmed their likely nationality, and the research assistants proceeded to explain the study. The first contact person was then asked to suggest a well-connected Myanmar migrant worker who could serve as an initial seed. This informal referral process ensured that the seeds were individuals who were trusted within their communities and had strong social networks, maximizing the effectiveness of the RDS process.

To address potential stigma and social bias, several measures were implemented:

- **Waived Consent and Anonymity:** Oral consent was obtained without collecting signatures or personal identifiers to alleviate concerns related to legal or undocumented status.
- **Confidentiality Assurance:** Participants were assured that all responses would remain strictly confidential and anonymous. Research assistants reinforced trust by reiterating, “Only you and I will know this,” without coercion.
- **Flexible Interview Settings:** Interviews were conducted on Sundays or after working hours in participants’ homes to ensure privacy and comfort. Interviews proceeded with family members present only if the participant expressed no objection.
- **Non-Threatening Approach:** Questions focused on personal and family life rather than workplace-related aspects to avoid fears tied to employment status.
- **Researcher Training:** Research assistants received training on sensitive questioning techniques and ethical considerations to ensure a non-judgmental approach.

### Study setting

This study was conducted in Southern Thailand (Songkhla and Surat Thani provinces), key destinations for Myanmar migrant workers in fisheries, manufacturing, and agriculture. We applied Thailand’s standard administrative-boundary method for urban-rural classification: urban areas comprised municipalities and special administrative zones (Bangkok, Pattaya), while other areas were classified as rural [33,34]. This approach effectively reflects structural and socioeconomic disparities between central and peripheral regions [34]. Participants’ residential areas were classified based on this criterion to contextualize environmental influences.

### Research instrument and validity

#### Instrument development and domains.

Eligible participants were invited to complete an 85-item structured questionnaire developed based on the Social Determinants of Health (SDH) framework and prior literature [28,29,35,36]. As shown in the conceptual framework in the supporting information, the survey assessed seven key domains: (1) alcohol consumption patterns (10 items), (2) environmental factors (22 items), (3) interpersonal relationships (4 items), (4) labor characteristics (8 items), (5) health behavior and status (8 items), (6) demographic and socioeconomic factors (8 items), and (7) alcohol-related impacts (25 items). This comprehensive approach ensures alignment with the SDH framework, which emphasizes multifactorial influences on health outcomes.

#### Independent variables and measures.

- **Alcohol consumption patterns**

Alcohol use was measured using the Alcohol Use Disorders Identification Test (AUDIT), a 10-item screening tool [36]. Items assessed consumption frequency (e.g., “How often do you consume alcohol?”), dependence symptoms, and harmful behaviors, rated on a 5-point Likert scale (0 = never, 4 = four or more times weekly). Total scores (0–40) were categorized as low-risk (0–7), hazardous (8–15), harmful (16–19), and probable dependence (≥20). For analytical robustness, hazardous and harmful categories were merged into a “risky drinkers” group (scores 8–19). It should be noted that while the AUDIT is widely validated globally [37], no formal validation in the Myanmar language exists.

- **Environmental factors**

Housing-related factors (12 items): This domain comprised seven checklist items (e.g., type of community: urban, rural, semi-urban/rural, migrant worker community) and five items rated on a 5-point scale (e.g., “Does your housing have overcrowding issues?”; 1 = none to 5 = high). Total scores ranged from 8 to 40, categorized as minor (8–18.66), moderate (18.67–29.33), or significant issues (29.34–40) [37].

Work environment factors (10 items): It comprised one checklist item, which identified the general type of workplace (e.g., outdoor, indoor, or mixed environments), and nine subsequent items rated on a 5-point Likert scale (1 = none to 5 = high) to assess specific environmental challenges. These challenges included noise, unpleasant odors, insufficient lighting, poor air circulation, dust, cold temperature, chemical exposure, prolonged water exposure, and overcrowding. For instance, participants responded to questions such as “Does your workplace have noise problems?” and “Does your workplace have problems with overcrowding?”. Total scores for this domain ranged from 9 to 45, with higher values indicating more challenging work environments, and these scores were then categorized as minor (9–21), moderate (22–38), or challenging (39–45) [38].

- **Interpersonal relationships**

Four single-item measures were used to assess relationship quality, evaluating connections with neighbors, workmates, family, and employers (e.g., “How is your relationship with your neighbors?”). Participants rated each item on a three-point Likert scale as poor, neutral, or good. For clarity, these response options were defined as follows:

Poor (1): Defined as a strained or conflictual relationship, characterized by a lack of communication, support, or trust.

Neutral (2): Indicated an average or functional interaction, without strong positive or negative sentiments.

Good (3): Reflected a positive, supportive, and trusting connection, involving mutual communication and assistance.

This simplified approach was chosen to reduce participant burden and ensure feasibility in the field, particularly given the linguistic and cultural diversity of Myanmar migrant workers. These items were selected based on critical social determinants identified as relevant to migrant populations. Although formal reliability testing was not conducted for these single-item measures, their validity and appropriateness were ensured through the processes described in the “Validity and reliability assessment” section.

- **Labor characteristics, health behaviors, and demographics**
  - Labor characteristics (8 items): Included province of residence, work duration, migrant status, health insurance, working hours/days, part-time employment, and annual health checkups.
  - Health behavior and status (8 items): Covered meal frequency, exercise, sleep disturbances, tobacco use, physical health status, chronic diseases, and health information receipt (available in Myanmar language).
  - Demographic and socioeconomic factors (8 items): Assessed gender, age, marital status, education, occupation, income, expenditure, and financial status.

#### Dependent variable: alcohol-related impacts.

The dependent variable was alcohol-related impacts, defined as self-reported negative consequences attributed to the respondent’s own alcohol consumption across three domains: health (e.g., injuries, illnesses), family (e.g., domestic conflict, violence), and social life (e.g., legal issues, community complaints). Examples of items include:

- Health domain: “How often have you experienced accidents in your daily life due to your drinking?”
- Family domain: “How often have you engaged in physical fights with family members because of your alcohol consumption**?”**
- Social domain: “How often have you received complaints from neighbors/community members about your behavior when drinking**?**”

A total of 25 items measured experiences over the past month using a 3-point frequency scale (1 = never/rarely, 2 = occasionally, 3 = frequently). Total scores ranged from 25 to 75, with higher scores indicating greater severity. Based on Kiess and Green [38], scores were initially categorized as low (25–41.66), moderate (41.67–58.33), and high impact (58.34–75). Due to limited high-impact cases (n = 5, 0.82%), moderate and high categories were merged into a ‘moderate-high impact’ group (≥41.67) to enhance statistical power in regression analyses [38].

#### Validity and reliability assessment.

- Content validity: Confirmed by five experts, with an Item–Objective Congruence Index (IOC) ranging from 0.80 to 1.00 [39].
- Reliability: Cronbach’s alpha values indicated acceptable to high internal consistency: 0.74 (housing factors), 0.85 (work environment factors), and 0.78 (alcohol-related impacts). The AUDIT subscale demonstrated high sensitivity (0.95) and specificity (0.80) in prior studies [36].
- Translation and cultural adaptation: The entire questionnaire was subjected to a rigorous forward-backward translation process (Thai-Burmese) to ensure linguistic and conceptual appropriateness for Myanmar migrant workers. This process also helped mitigate potential limitations, such as the lack of a formally validated AUDIT instrument in Myanmar. The translated instrument was subsequently pilot-tested with 30 participants to refine item clarity and cultural relevance.

### Data collection procedures

Data were collected between September 1, 2023, and March 31, 2024, following approval from the Provincial Public Health Offices. Three trained bilingual interpreters (Myanmar nationals fluent in Thai) conducted structured, paper-based interviews at participants’ residences using questionnaires in Thai and Burmese. This study received approval from the Research Ethics Committee of Rajabhat University (Approval No: SRU-EC2023/119, dated 11 September 2023). Oral consent was obtained from participants to safeguard vulnerable migrant workers, particularly those with undocumented status, by ensuring confidentiality and alleviating legal concerns.

Before participation, the study objectives, procedures, potential risks, and their right to decline or withdraw without consequence were clearly explained, ensuring participants made an informed decision. Individuals who opted not to participate received the same treatment as those who did. Verbal agreement or a nod confirmed consent. Only individuals who voluntarily agreed to participate were interviewed. Upon completion, each participant received a culturally appropriate token of appreciation (a towel valued at 50 THB), as approved by the ethics committee.

### Statistical analysis

Descriptive statistics were used to summarize the characteristics of the study population. Simple logistic regression was used for bivariate analysis to assess the association between independent variables and the impact of alcohol consumption, categorized as low vs. moderate/high. Variables of interest—including alcohol use, environmental factors, and other covariates with p-values < 0.25—were included in multivariable analysis using a Generalized Linear Mixed Model (GLMM), accounting for provinces as random effects to identify factors linked to alcohol-related impacts [40]. Multicollinearity was assessed with a variance inflation factor (VIF) threshold of <10. Model fitting employed backward elimination, with statistical significance set at p < 0.05. An adjusted odds ratio (Adj. OR) of 1 indicated no association, > 1 indicated a risk factor, and <1 indicated a protective effect [40]. Data analysis was conducted using Stata 18.0 BE, a software package licensed by Khon Kaen University.

## Results

Descriptive statistics showed a complete dataset with no missing data for the 610 participants. Of these participants, the majority were male (74%) with a mean age of 35 years. Sociodemographically, most were married (58%), nearly one-third (31%) had completed lower secondary education, and the primary employment sector was the fishing industry (39%). Economically, the median monthly personal income was 10,000 THB, double the median expenses (5,000 THB); however, a significant majority (76%) reported having no savings. Regarding living and working conditions, most participants (53%) resided in a Myanmar migrant worker community, with the majority reporting low environmental problems in housing (90%). Most worked ≤8 h/day (76%) and ≤6 days/week (83%). In health and wellbeing, prevalent concerns included sleep problems (58%), though most reported good physical health (62%). Nearly half (47%) reported a moderate relationship with co-workers. Concerning alcohol use, the majority of participants (73%) were classified as low-risk drinkers (AUDIT scores 0–7). Notably, 14% met the criteria for probable alcohol dependence (scores ≥20). Furthermore, 6% were hazardous drinkers (scores 8–15) and 6% were harmful drinkers (scores 16–19). Detailed characteristics are provided in the supporting information.

Table 1 presents the distribution of impact levels from alcohol consumption. Most respondents (61%) reported low impact, while 38% reported moderate impact. Only a negligible proportion (<1%) reported a high impact. The mean impact score was 37 (SD = 9.6), with a median of 36 (range: 25–73).

**Table 1:** Number, percentage, 95% CI, and levels of impact from alcohol consumption (*n* = 610).

Issues	Number	Percentage	95% CI
**Level of impact from alcohol consumption**			
Low (25–41.66 points)	372	60.98	57.04-64.78
Moderate (41.67–58.33 points)	233	38.20	34.41-42.12
High (58.34–75 points)	5	0.82	0.34-1.95
Mean (Standard deviation)	36.96 (9.64)		
Median (Min-Max)	36 (25-73)		

Results from the regression analysis (Table 2) identified several key factors associated with increased odds of alcohol-related health, family, and social problems. Both alcohol addiction and risky drinking demonstrated significant associations, with adjusted odds ratios (AORs) of 1.70 and 1.65, respectively, compared to low-risk drinkers. Rural residence was the strongest correlate, with an adjusted odds ratio of 6.52 compared to urban communities, while Myanmar worker communities had an adjusted odds ratio of 1.31. Substantial associations were also observed for individuals reporting moderate or high housing issues (AOR = 5.00) and those working over eight hours daily (AOR = 2.39). Furthermore, poor sleep quality (AOR = 2.09), moderate/poor health (AOR = 2.11), and poor relationships with co-workers (AOR = 1.89) were all significantly associated with higher reporting of these multifaceted problems.

**Table 2:** Association between alcohol consumption, environment, and outcome variables (n = 610).

Factors	Number	% Moderate-high impact	Crude OR	Adj. OR	95% CI	p value
Level of alcohol consumption and environmental factors						
**Level of alcohol consumption**						0.020
Low-risk drinkers	446	34.30	1	1	1	
Risky drinkers	76	38.16	1.18	1.65	1.08-2.52	
Dependent drinkers	88	63.64	3.35	1.70	1.61-1.79	
**Type of community**						< 0.001
Urban community	148	11.49	1	1	1	
Myanmar workers community	323	40.25	5.19	1.31	1.20-1.42	
Rural community or other	139	65.47	14.60	6.52	4.35-9.77	
**Environmental problems in housing**						< 0.001
Low	548	34.49	1	1	1	
Moderate/high	62	79.03	7.15	5.00	2.70-9.24	
Other covariates						
**Period of work (hours per day)**						0.044
≤ 8	480	30.83	1	1	1	
> 8	130	69.23	5.04	2.39	1.02-5.60	
**Amount of time to work (day/week)**						<0.001
≤ 6	521	33.01	1	1	1	
Everyday	89	74.16	5.82	2.39	1.64-3.49	
**Poor sleep quality**						<0.001
No	259	25.10	1	1	1	
Yes	351	49.29	2.90	2.09	1.51-2.90	
**Health**						<0.001
Strong	376	25.80	1	1	1	
Moderate/poor	234	60.26	4.36	2.11	2.02-2.22	
**Relationships with co-workers**						<0.001
Good	275	30.91	1	1	1	
Moderate/poor	335	45.67	1.87	1.89	1.88-1.90	

To further support the use of GLMM in this analysis, the estimated standard deviation of the province-level random intercept was 0.79 (variance = 0.63). The corresponding intraclass correlation coefficient (ICC) was 0.16 (95% CI: 0.12–0.22), indicating that approximately 16% of the total variability in the outcome was attributable to differences at the province level.

- Community types:
  - Myanmar workers community: Areas primarily inhabited by Myanmar migrant workers, often near workplaces or based on shared nationality/culture.
  - Urban community: Residential areas within towns or cities, characterized by high density, diverse economy, and developed infrastructure.
  - Rural community: Geographically less developed areas, often agricultural or transitional zones between urban and rural settings.
- Environmental problems in housing referred to various adverse conditions within participants’ residences impacting health and well-being. These included issues related to sanitation (e.g., unpleasant odors, garbage problems), indoor environmental quality (e.g., poor air circulation, insufficient lighting, dust), noise exposure, and overcrowding.

## Discussion

This study offers comprehensive analysis of multi-faceted impacts of alcohol consumption among Myanmar migrant workers in Thailand. It is important to first clarify that our outcome measure, ‘alcohol-related impacts,’ captured self-reported negative consequences specifically attributed by respondents to their alcohol consumption across three domains: health (e.g., injuries, illnesses), family (e.g., conflicts, violence), and social (e.g., legal issues, community complaints).

This population experiences unique vulnerabilities due to their socio-legal status, cultural displacement, and concentration in high-risk industries. The study moves beyond general knowledge by demonstrating how these migration experiences shape alcohol-related outcomes, specifically by examining the relationship between alcohol dependence, housing conditions, and community context. Our findings highlight the substantial role of contextual factors, particularly rural residence and poor housing, which showed stronger associations with negative outcomes than alcohol dependence severity in this population. This underscores that the problem is rooted more in environmental vulnerabilities than individual addiction levels.

The study revealed that approximately two-fifths of the sample experienced a moderate to severe alcohol-related impact. This high prevalence aligns with previous findings on the negative outcomes of drinking among migrant workers [41]. These impacts are multifaceted. For instance, mental health issues such as depression, anxiety, and substance dependence are common yet often neglected due to limited healthcare access and stigma. Economically, alcohol misuse undermines stability by reducing productivity, increasing the risk of job loss, and lowering remittances to families [42]. In addition, it raises the risk of workplace accidents, thereby compounding existing health and financial vulnerabilities [41].

Our analysis confirmed that alcohol dependence was associated with a 1.70 times higher likelihood of reporting alcohol-related consequences compared to low-risk drinkers. These reported consequences were strongly correlated with adverse health conditions, impaired family relationships, and negative social consequences. This aligns with previous findings from Myanmar and Nepal, where heavy drinking has been linked to violent incidents and domestic abuse, especially among women whose spouses consume alcohol regularly [43,44]. Additionally, high-risk drinkers had 22 times more work absences than low-risk drinkers [45].

This study newly demonstrates that rural settings were the strongest alcohol-related correlate, surpassing individual factors like addiction severity. This contrasts with literature emphasizing individual patterns and highlights this population’s unique socio-ecological context [27,46]. Data show that migrants in rural areas experienced these impacts more frequently than their urban counterparts. This disparity stems from a confluence of factors specific to their marginalization: evidence suggests rural residents tend to drink more, increasing risks of liver disease, mental health problems, and injuries [25,47].

The residential environment was significantly associated with outcomes and was the second strongest correlate. For migrant workers, the quality of housing is not merely a matter of comfort but a fundamental factor influencing mental well-being and coping behaviors. This finding extends previous research on housing and health by highlighting its disproportionate impact within a population that has little control over their living conditions, a situation less common in studies of housed non-migrant populations.

Workers in poor home environments faced higher risks than those in stable housing. Housing dissatisfaction and overcrowding are associated with higher alcohol use among migrants [29,48]. Substandard housing, characterized by limited space and poor quality, undermines personal control and causes chronic stress. These conditions link to depression, anxiety, which may lead to alcohol use as a maladaptive coping strategy [49,50]. Workplace stressors combined with poor housing predict problem drinking, as domestic pressures intensify work stress and elevate alcohol risks [51,52].

Individuals with limited workplace support may struggle to address alcohol-related challenges effectively [53]. These factors were exacerbated by demanding conditions like long working hours (>8 daily) and no rest days, which showed a strong association with alcohol problems, aligning with findings from South Korea and Norway [35,54]. Demanding schedules increase stress and fatigue, limiting rest and social support, thereby promoting alcohol use as a coping strategy [55]. This then aggravates alcohol-related impacts across health, including alcohol use disorders and mental/physical illnesses; family, through work–family conflict; and social domains, leading to isolation [55–57].

Individual health behaviors were also significant. Participants with poor sleep quality reported more alcohol-related impacts, aligning with evidence linking heavy drinking to sleep disturbances [58,59]. The alcohol-physical illness interplay proves especially detrimental for those with mental health conditions, worsening their overall health burden [60]. Compounding lifestyle factors, particularly smoking and physical inactivity, further drive this dynamic [6]. In our sample, one-third of workers exhibited multiple health risks simultaneously: physical health problems, tobacco use, physical inactivity, and inadequate sleep.

Interpersonal dynamics in the workplace also played a key role. Poor or moderate relationships with co-workers were correlated with increased vulnerability to alcohol-related impacts. Prior research shows that negative workplace relationships can lead to elevated stress, low job satisfaction, and poor organizational commitment [61], which may drive alcohol use as a coping mechanism.

These findings align with the Social Determinants of Health framework, where health outcomes are shaped by both individual behaviors and broader social conditions. Contextual disadvantages, particularly rural residence and poor housing, were more strongly associated with alcohol-related impacts than alcohol dependence among Myanmar migrant workers. Limited healthcare access, economic insecurity, and social isolation may exacerbate alcohol’s adverse consequences. Alcohol may serve as a coping mechanism for stress, while inadequate structural support amplifies its detrimental effects on health, family, and social well-being [27,49]. These findings, therefore, carry significant public health implications, underscoring the necessity for integrated, multi-sectoral public health strategies that prioritize addressing living and environmental conditions beyond individual behaviors.

International organizations such as the World Health Organization (WHO), the United Nations High Commissioner for Refugees (UNHCR), and the International Organization for Migration (IOM) play a vital role in addressing these challenges by advocating for improved living and working conditions, equitable healthcare access, and culturally tailored health education. Their efforts can promote safe housing, fair labor practices, mental health support, and alcohol risk awareness to support vulnerable migrant communities.

This study provides vital public health insights for enhancing migrant workers’ health, offering practical implications for interventions and policy. Longitudinal research is necessary to determine causal relationships between workplace stressors, housing conditions, and alcohol use. In addition, future studies should consider follow-up data collection and in-depth qualitative research to better understand the dynamics of alcohol use behavior and its associated factors in this population. These public health implications include practical interventions like peer support groups, better housing, and trained workplace health volunteers to mitigate alcohol-related impact. Furthermore, policies are needed to emphasize safe, hygienic housing and work-life balance, while public health education should target alcohol risks and promote healthy coping strategies through employer-led support.

Key strengths include the focus on an underrepresented population and the use of culturally sensitive data collection, which enhanced trust and data accuracy. Despite these strengths, it is important to acknowledge the limitations of this study. The cross-sectional design precludes causal inference, and the findings may not be generalizable to all migrant groups. Furthermore, this study may be subject to recall bias, as the data on alcohol consumption and its related impacts were based on self-reports.

## Conclusions

This study demonstrates that alcohol-related impacts among Myanmar migrant workers in Thailand are driven more by adverse environmental and social conditions than by individual drinking patterns. Approximately two-fifths of workers reported moderate to severe alcohol-related impacts. These outcomes were more strongly associated with upstream factors like rural residency and housing problems than with alcohol dependence itself. Other significant contributors included excessive work hours, physical health problems, sleep disturbances, and poor workplace relationships.

These results underscore the necessity for integrated public health strategies that address both alcohol use and its structural drivers. Improving living and working conditions, along with culturally adapted health promotion, is essential for reducing alcohol-related impacts in this vulnerable population.

## Supporting information

S1 Fig Alcohol consumption levels among Myanmar migrant workers in Southern Thailand (n = 610). (TIF)

S2 Fig Conceptual framework of factors associated with alcohol-related impacts among Myanmar migrant workers. (TIF)

S1 Table Characteristics of the study participants (n = 610). (DOCX)

S2 Table Number and percentage of health behavior, physical health, and health information (n = 610). (DOCX)

S3 Table Numbers and percentages classified by environment and relationship factors (n = 610). (DOCX)
