# Supplementary material for: Alcohol use and environmental factors: A cross-sectional study exploring health risks and social implications among Myanmar migrant workers
Source: PLoS One. 2026 Mar 5;21(3):e0343825. doi: 10.1371/journal.pone.0343825 (PMC12962502; doi:10.1371/journal.pone.0343825)
Supplement: S1 Table — (DOCX) [file pone.0343825.s003.docx]

**Supporting information**

**S1 Table. Characteristics of the study participants (n = 610).**

This table provides a detailed profile of the study participants, including their age, sex, marital status, education, occupation, income, and other key demographic and economic indicators.

| **Demographic and socioeconomic characteristics** | **Number** | **Percentage** |
| --- | --- | --- |
| **Sex** |  |  |
| Female | 159 | 26.07 |
| Male | 451 | 73.93 |
| **Age (years)** |  |  |
| ≤ 19 | 18 | 2.95 |
| 20–29 | 187 | 30.66 |
| 30–39 | 239 | 39.18 |
| 40–59 | 147 | 24.10 |
| ≥ 60 | 19 | 3.11 |
| Mean (Standard deviation) | 34.80 (10.61) | |
| Median (Min–Max) | 33 (18–73) | |
| **Marital status** |  |  |
| Single | 208 | 34.10 |
| Married (with a marriage certificate) | 217 | 35.57 |
| Married (without a marriage certificate) | 136 | 22.30 |
| Divorced/widowed/separated | 49 | 8.03 |
| **Highest education level** |  |  |
| No formal education | 33 | 5.41 |
| Primary school | 180 | 29.51 |
| Lower secondary school | 187 | 30.66 |
| High school or equivalence | 153 | 25.08 |
| Diploma or equivalence | 44 | 7.21 |
| Bachelor’s degree or higher | 13 | 2.13 |
| **Main occupational group** |  |  |
| Fishing sector | 235 | 38.52 |
| Service industry sector | 153 | 25.08 |
| Agriculture and animal feeding sector | 114 | 18.69 |
| Construction sectors | 43 | 7.05 |
| Household | 39 | 6.40 |
| Manufacturing industry sector | 7 | 1.15 |
| Others | 19 | 3.11 |
| **Average monthly income (baht/month)** |  |  |
| None | 15 | 2.46 |
| < 7,500 | 37 | 6.07 |
| 7,500–9,999 | 224 | 36.72 |
| 10,000–12,499 | 227 | 37.21 |
| ≥ 12,500 | 107 | 17.54 |
| Mean (Standard deviation) | 10,195.41 (3,169.76) | |
| Median (Min–Max) | 10,000 (0–21,000) | |
| **Average monthly expenditure (baht/month)** |  |  |
| None | 15 | 2.46 |
| < 2,500 | 35 | 5.74 |
| 2,500–4,999 | 110 | 18.03 |
| 5,000–7,499 | 355 | 58.19 |
| ≥ 7,500 | 95 | 15.67 |
| Mean (Standard deviation) | 5,457.37 (2,099.68) | |
| Median (Min–Max) | 5,000 (0–12,000) | |
| **Financial status** |  |  |
| Not enough and debt | 94 | 15.41 |
| Not enough and not debt | 143 | 23.44 |
| Enough but not saving | 225 | 36.89 |
| Enough and saving | 148 | 24.26 |
| **Live in province** |  |  |
| Songkhla | 240 | 39.34 |
| Surat Thani | 370 | 60.66 |
| **Worked in Thailand (years)** |  |  |
| < 2 | 100 | 16.39 |
| 2–5 | 234 | 38.36 |
| 6–10 | 161 | 26.40 |
| > 10 | 115 | 18.85 |
| Mean (Standard deviation) | 6.71 (6.30) | |
| Median (Min–Max) | 5 (0.08–36) | |
| **Status of migrant** |  |  |
| Illegal | 59 | 9.67 |
| Legal | 551 | 90.33 |
| **Health insurance** |  |  |
| No | 83 | 13.61 |
| Yes | 527 | 86.39 |
| **Working hours per day (hours)** |  |  |
| No job | 14 | 2.30 |
| ≤ 8 | 466 | 76.39 |
| > 8 | 130 | 21.31 |
| Mean (Standard deviation) | 8.18 (1.64) | |
| Median (Min–Max) | 8 (0–15) | |
| **Working days per week (days)** |  |  |
| No job | 14 | 2.30 |
| ≤ 6 | 507 | 83.11 |
| Everyday | 89 | 14.59 |
| Mean (Standard deviation) | 5.88 (1.10) | |
| Median (Min–Max) | 6 (0–7) | |
| **Worked part-time** |  |  |
| Never | 417 | 68.36 |
| Yes | 193 | 31.64 |
| **Annual health checkup during work** |  |  |
| Never | 161 | 26.39 |
| Yes | 449 | 73.61 |
