# Supplementary material for: Alcohol use and environmental factors: A cross-sectional study exploring health risks and social implications among Myanmar migrant workers
Source: PLoS One. 2026 Mar 5;21(3):e0343825. doi: 10.1371/journal.pone.0343825 (PMC12962502; doi:10.1371/journal.pone.0343825)
Supplement: S2 Table — (DOCX) [file pone.0343825.s004.docx]

**Supporting information**

**S2 Table. Number and percentage of health behavior, physical health, and health information (n=610).**

This table summarizes the prevalence of key health behaviors, self-reported physical health status, and the availability of health-related information to the participants.

| **Health behavior, physical health, and health information** | **Number** | **Percentage** |
| --- | --- | --- |
| **Meals per day in past month** |  |  |
| < 3 | 131 | 21.48 |
| 3 | 450 | 73.77 |
| > 3 | 29 | 4.75 |
| **Exercise in past month** |  |  |
| No | 227 | 37.21 |
| Yes | 383 | 62.79 |
| **Poor sleep quality** |  |  |
| No | 259 | 42.46 |
| Yes | 351 | 57.54 |
| **Tobacco used** |  |  |
| Non-smoker | 390 | 63.93 |
| Former smoker | 17 | 2.79 |
| Smoker | 203 | 33.28 |
| **Health** |  |  |
| Not strong | 5 | 0.82 |
| Moderate | 229 | 37.54 |
| Strong | 376 | 61.64 |
| **Chronic illness** |  |  |
| No | 511 | 83.77 |
| Yes | 99 | 16.23 |
| **Received health-related information** |  |  |
| No | 230 | 37.70 |
| Yes | 380 | 62.30 |
| **Received health information materials in Myanmar  language** |  |  |
| No | 174 | 28.52 |
| Yes | 436 | 71.48 |
