# Supplementary material for: Alcohol use and environmental factors: A cross-sectional study exploring health risks and social implications among Myanmar migrant workers
Source: PLoS One. 2026 Mar 5;21(3):e0343825. doi: 10.1371/journal.pone.0343825 (PMC12962502; doi:10.1371/journal.pone.0343825)
Supplement: S3 Table — (DOCX) [file pone.0343825.s005.docx]

**Supporting information**

**S3 Table. Numbers and percentages classified by environment and relationship factors (n=610).**

This table describes the housing and workplace settings, environmental challenges, and the quality of social relationships reported by the migrant workers.

| **Environment and relationship factors** | **Number** | **Percentage** |
| --- | --- | --- |
| **Type of community** |  |  |
| Myanmar workers community | 323 | 52.95 |
| Urban community | 148 | 24.26 |
| Rural community | 100 | 16.39 |
| Semi-urban, semi-rural community | 37 | 6.07 |
| Others | 2 | 0.33 |
| **Type of residence** |  |  |
| Worker camp | 447 | 73.28 |
| Shared rental house | 88 | 14.43 |
| Detached rental house | 66 | 10.82 |
| Apartment | 5 | 0.82 |
| Others | 4 | 0.66 |
| **Live with** |  |  |
| Family | 397 | 65.08 |
| Friend | 168 | 27.54 |
| Alone | 43 | 7.05 |
| Others | 2 | 0.33 |
| **Environmental problems in housing** |  |  |
| Low | 548 | 89.84 |
| Moderate | 57 | 9.34 |
| High | 5 | 0.82 |
| **Nature of workplace** |  |  |
| Outdoor | 213 | 34.91 |
| Both outdoor and indoor | 187 | 30.66 |
| Indoor | 182 | 29.84 |
| Others | 28 | 4.59 |
| **Environmental problems in workplace** |  |  |
| Low | 560 | 91.80 |
| Moderate | 50 | 8.20 |
| High | 0 | 0 |
| **Perceived relationship with** **neighbors** |  |  |
| Poor | 49 | 8.03 |
| Moderate | 308 | 50.49 |
| Good | 253 | 41.48 |
| **Perceived relationship with workmates** |  |  |
| Poor | 48 | 7.87 |
| Moderate | 287 | 47.05 |
| Good | 275 | 45.08 |
| **Perceived relationship with family** |  |  |
| Poor | 44 | 7.21 |
| Moderate | 219 | 35.90 |
| Good | 347 | 56.89 |
| **Perceived relationship with employer** |  |  |
| Poor | 52 | 8.52 |
| Moderate | 259 | 42.46 |
| Good | 299 | 49.02 |
